# Low incidence of COVID-19 in a prospective cohort of patients with liver cirrhosis and hepatocellular carcinoma treated at a tertiary medical center during the 2020 pandemic

**DOI:** 10.1371/journal.pone.0258450

**Published:** 2021-12-23

**Authors:** Thorben Fründt, Lilith Kuballa, Marc Lütgehetman, Dominik Nörz, Hannes Arend, Thomas T. Brehm, Julian Schulze zur Wiesch, Thomas Horvatits, Karoline Horvatits, Samuel Huber, Henning Wege, Johannes Kluwe

**Affiliations:** 1 Department of Medicine, University Medical Center Hamburg-Eppendorf, Hamburg, Germany; 2 Institute of Microbiology and Virology, University Medical Center Hamburg-Eppendorf, Hamburg, Germany; 3 Department of Internal Medicine, Israelitic Hospital, Hamburg, Germany; 4 German Center for Infection Research (DZIF), Partner Site Hamburg-Lübeck-Borstel-Riems, Braunschweig, Germany; 5 Department of Medicine, Hematology, Oncology, Gastroenterology and Infectious Diseases, Klinikum Esslingen, Esslingen, Germany; Medizinische Fakultat der RWTH Aachen, GERMANY

## Abstract

**Background and aims:**

Patients with liver cirrhosis (LC) are considered to be at increased risk for mortality when acquiring SARS-CoV-2 infection and subsequently developing Corona Virus Disease 2019 (COVID-19). During the COVID-19 pandemic, hospitals are regarded as sites with increased risk of infection. Therefore, patient contacts are often limited to urgent indications, which could negatively affect disease monitoring. However, data regarding actual infection rates in cirrhotic patients is limited. The aim of this prospective study was to assess the incidence of COVID-19 in patients with LC with/without hepatocellular carcinoma (HCC) with physical presentation at our University Medical Center.

**Methods:**

Patients were enrolled between 1st April and 30th June 2020 at the University Medical Center Hamburg-Eppendorf, Germany. Symptoms of upper airway infection at baseline and presence of SARS-CoV-2 antibodies (IgG/IgM/IgA) were assessed at baseline and follow-up (FU) using an Electro-chemiluminescence immunoassay (Roche Elecsys). FU visits, including liver function test, clinical assessment and symptom questionnaire, were conducted after 6–8 weeks (FU-1) and 6 months (FU-2). Prior to inclusion of the first patient, obligatory face masks and personal distance were implemented as protective measures.

**Results:**

A total of 150 patients were enrolled, 23% (n = 35) also had diagnosis of HCC (median age: 64 years, range: 19–86), 69% were male. Liver function according to Child-Pugh score (CPS) was: CPS A: 46% (n = 62); CPS B: 37% (n = 50); CPS C: 17% (n = 23). Clinical symptoms indicating upper airway infection were present in 53% (n = 77): shortness of breath (n = 40) and coughing (n = 28) were the most frequent. For the 150 patients enrolled, 284 outpatient visits were registered and 33 patients were admitted to the University Medical Center during the follow-up period. After a median of 52 days, n = 110 patients completed FU-1 and n = 72 completed FU-2 after a median of 6.1 months. Only in one patient, an 80-year-old man with stable liver function (CPS A) and advanced HCC, SARS-CoV-2 antibodies were detected at baseline and FU-1, while antibody testing was negative in the remaining patients at baseline, FU-1 and FU-2.

**Conclusion:**

The incidence of COVID-19 at our tertiary medical center during the pandemic was low in LC and HCC patients, when simple protective measures were implemented. Therefore, a routine care for patients with chronic liver diseases does not increase the risk of SARS-CoV-2 infection and should be maintained with protective measures.

## Introduction

Beginning in the city of Wuhan, China, at the end of 2019, the new severe acute respiratory syndrome coronavirus 2 (SARS- CoV-2) caused a pandemic that spread around the world and had soon been declared by the WHO as a Public Health Emergency of International Concern on 30 January 2020 [1, 2]. As of 28th August 2021, more than 215 million people had been infected worldwide leading to over 4.400.000 reported casualties so far [3]. People who are infected with SARS-CoV-2 often develop COVID-19, characterized by flu like symptoms such as fever, cough, sore throat and headache [4]. Approximately 10% of these patients’ progress to severe hypoxemia and pneumonia and partially develop acute respiratory distress syndrome (ARDS), resulting in a mortality rate ranging from 2% up to 7% [4, 5]. Several risk factors for severe clinical course and COVID-19 associated mortality and death have been described, including cancer, hypertension, coronary heart disease, obesity and older age [6–9]. Recently, patients with liver cirrhosis were reported to be at risk for increased mortality and deterioration of liver function following COVID-19 [10]. In addition, Marjot et al. demonstrated a stepwise increase in mortality with worsening liver function in patients with liver cirrhosis and COVID-19 [11]. Nosocomial transmission of SARS-CoV-2 has been reported and face to face contact as well as presentation to the healthcare system have been identified as risks factors for virus transmission [12, 13]. With respect to the vulnerability of patients with liver cirrhosis and liver cancer, it is a tremendous challenge to find a balance between sufficient medical care and disease surveillance for these patients on the one hand, and prevention of SARS-Cov2-transmission during presentation at healthcare facilities on the other hand.

The Center of Disease Control recommends face masks for health care practitioners as well as for patients, to keep physical distance while at the outpatient department and to schedule appointments in a distinct manner, so that only a limited number of patients are present in the waiting room at the same time [14]. For patients with liver cirrhosis (LC) or hepatocellular carcinoma (HCC), the European Association for the Study of Liver (EASL) suggested a stratified outpatient care depending on the patients’ liver function (compensated or decompensated liver cirrhosis) in order to avoid putting vulnerable patients at risk for nosocomial infection [15].

Infection control interventions and stratified preventive measures for patient contacts are doubtlessly necessary. However, overcautious restrictions may negatively impact medical care and outcome of patients with liver cirrhosis and/or HCC. Real-life data on nosocomial SARS-CoV2-transmissions for patients with liver cirrhosis and HCC are lacking, but urgently needed for a valid risk-benefit-assessment.

Therefore, the aim of this prospective cohort study was to assess the incidence of COVID-19 infection in patients with LC and/or HCC who were treated at a tertiary referral center during the ongoing SARS-CoV-2 pandemic.

## Material and methods

### Study design and rational

We conducted a prospective cohort study to assess the rate of SARS-CoV-2 infection in patients with LC and/or HCC. The study protocol was reviewed and approved by the Ethics Committee of the Medical Council of Hamburg (PV 7298). Patients with LC and HCC patients with underlying liver cirrhosis without a history of previous COVID-19, who presented to the outpatient department or who were treated as inpatients were recruited at the University Medical Center Hamburg-Eppendorf between 1st April and 30th June, 2020. Diagnosis of liver cirrhosis was based on histology or on significantly elevated liver stiffness measured by transient elastography (Fibroscan^®^), using a cut-off value of > 14.6 kPa for diagnosis of liver cirrhosis as previously described [16]. HCC was diagnosed histologically or by imaging criteria according to recent guidelines [17]. On enrolment, all patients were tested for the presence of anti-SARS-CoV-2 antibodies. Additionally, all inpatients had been negatively tested for SARS-CoV-2 infection via nasopharyngeal swab and PCR within three days prior to admission. Liver function was assessed using the Child-Pugh score (CPS) and the Model for End-Stage Liver Disease (MELD), HCC was staged according to Barcelona Clinic Liver Cancer (BCLC) classification. The Charlson Comorbidity Index was calculated to assess morbidity and mortality in all patients included. First follow up (FU-1) visit was conducted six to eight weeks after enrolment between 20^th^ May and 24^th^ August. The second follow-up (FU-2) was scheduled six to eight months after baseline between 1^th^ October to 29^th^ January 2021 at the outpatient department of the University Medical Center Hamburg-Eppendorf. Serological testing for SARS-CoV-2, routine laboratory and clinical assessment of liver function tests including CPS and MELD-score, as well as standardized assessment of clinical symptoms were performed at baseline and repeated at every follow-up visit.

### Data collection and variables

Upon study inclusion, demographic and clinical data, laboratory testing including CPS and MELD score and tumor stage according to BCLC classification were assessed. Decompensated liver cirrhosis was diagnosed when ascites, hepatic encephalopathy, hepatorenal syndrome or variceal bleeding were present. Ascites was diagnosed either by ultrasound, by CT scan or by MRI. Hepatic encephalopathy as well as hepatorenal syndrome were diagnosed according to recent guidelines [18–20].

At enrolment, all patients were questioned for recent contact to persons with known COVID-19 disease using a structured questionnaire. Also, clinical symptoms at study inclusion and symptoms that had developed or had been present during the past four weeks including fever, cough, dyspnoea, rhinorrhoea, sore throat, headache, stomach pain, joint and muscle pain, nausea and diarrhoea were registered. Additionally, patient’s profession, travel activities within the past four weeks, number of household residents, smoking and drinking habits were recorded.

### Description of routine preventive measures at the study site

At the University Medical Center Hamburg-Eppendorf, preventive measures were implemented according to recommendations of a local COVID-19 task force, which includes experts in the field of virology, hospital hygiene, infectious diseases and intensive care medicine, established in February 2020. The following preventive measures to prevent SARS-CoV2-transmission at the University Medical Center Hamburg-Eppendorf were implemented during the observational period pandemic (Table 1): mandatory wearing of face masks at the emergency department since March 18; visitor restriction at the Medical Center since March 19; mandatory wearing of face masks in all clinical areas since March 22, mandatory wearing of filtering face piece (FFP) type 2 masks at the Department of Oncology since April 10, and PCR-based screening for SARS-CoV-2 within 5 days prior to admission or on admission in all patients since April 20. Doctors and nurses in direct contact to COVID-19 patients were tested on regularly basis since May 11.

**Table 1:** Chronological listing of the preventive measures, local and national incidence of SARS-CoV-2. The first patient of this study was enrolled on 2 April 2020.

Date	Preventive measures	SARS-CoV-2 incidence (n/d)	
		Hamburg	Germany
11 March	Annulation of non-patient care related meetings	83	2683
18 March	Mandatory wearing of face mask in the ER	133	4330
19 March	Visitor restriction for the entire medical center	89	3771
22 March	Mandatory wearing of face masks in all clinical departments	79	2947
10 April	Mandatory wearing of FFP 2 masks in the department of haematology and oncology	40	1615
20 April	PCR-based patient screening on admission	20	1039
11 May	PCR-based screening of medical stuff carrying for Covid-19 patients	6	374

Abbreviations: ER; emergency department; FFP 2, filtering facepiepe type 2.

### SARS-CoV-2 assay

Anti-SARS-CoV-2 antibodies were detected using the Roche Elecsys^®^ Anti-SARS-CoV-2 assay, a quantitative electrochemiluminescence immunoassay (ECLIA), detecting pan-immunoglobins (IgA, IgM and I directed against SARS-CoV-2 nucleocapsid protein). The assay is reported to have 96.7% sensitivity and 100% specificity [21]. All inpatients were tested for SARS-CoV-2 infection via nasopharyngeal swab prior to or on admission to hospital. In patients who had been diagnosed positive for SARS-CoV-2 antibodies, the DiaSorin Liaison^®^ assay, an assay detecting IgG-antibodies direct against the SARS-CoV-2 spike protein 1 (S1) and 2 (S2), was used to very test results.

### Statistical analysis

Continuous variables were described as median with minimum/maximum and range. For categorical variables, absolute and relative numbers were presented. Continuous variables were compared using the Mann-Whitney U test and categorical variables were compared using chi-square test. Results were subjected to statistical analysis using GraphPad Prism 8.3.0 (GraphPad San Diego, CA) and Excel 2019 (Microsoft). A two-sided P value less than 0.05 was considered significant.

## Results

**Fig 1:**
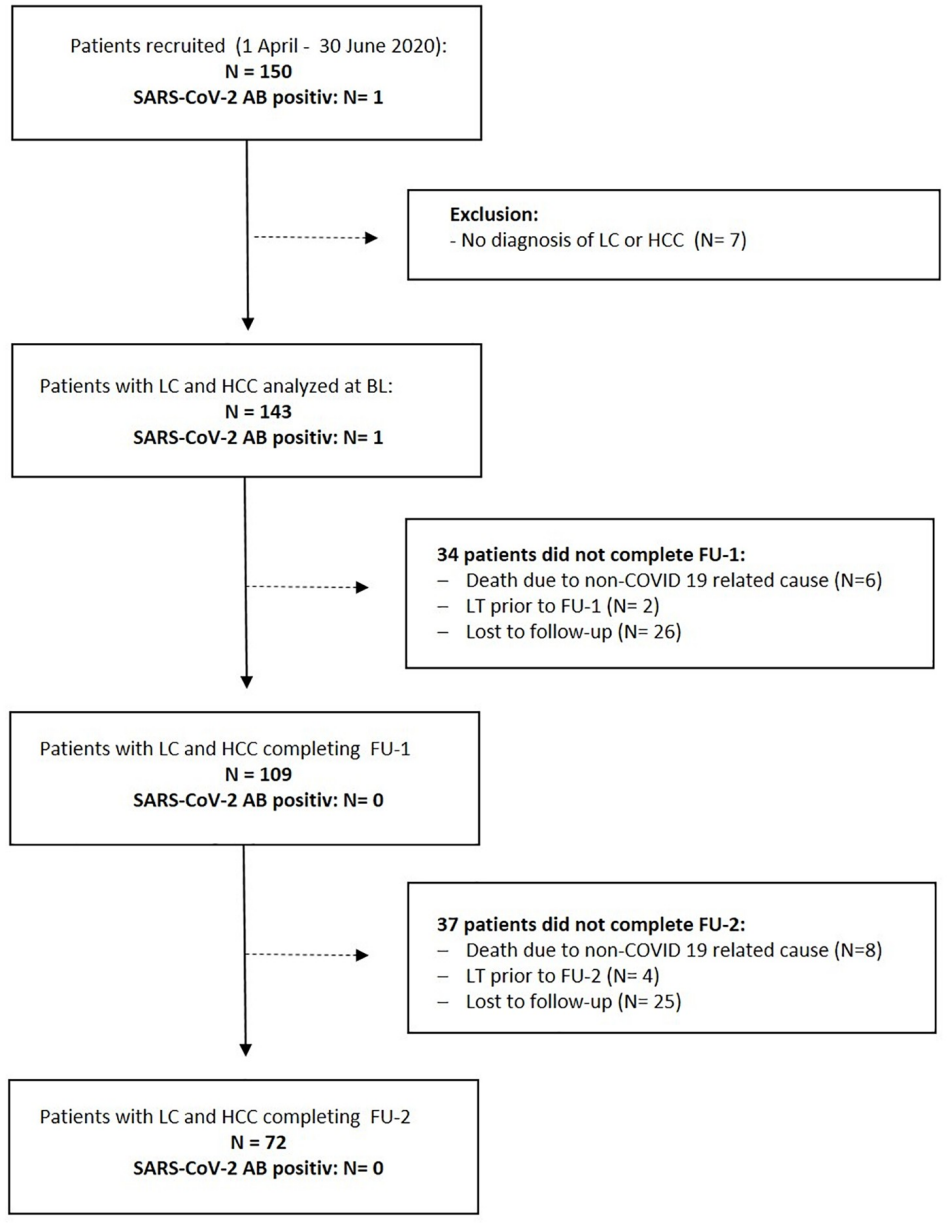
Flow chart of the study. FU-visits were conducted six to eight weeks (FU-1) and six months (FU-2) after enrolment. Since patients were only tested in the context of routine presentations in the outpatient department, a total of 51 patients did not complete FU-1 or FU-2, no routine presentation was indicated. Legend: HCC: hepatocellular carcinoma; LC: liver cirrhosis; LT: liver transplantation.

A total 150 patients were prospectively enrolled, 69% were male, median age was 59 years, range 19–85 years. A flow-chart of the study procedure is depicted in Fig 1. After enrolment, seven patients were excluded from further analysis, because neither LC nor HCC was present, n = 143 were analysed at baseline (BL). Liver cirrhosis was diagnosed histologically in 47% of all patients. Liverstiffness (median, min/max) according to Fibroscan measurement was 40.3 kPa (17.8-75kPa) in patients with alcoholic liver disease, 37 kPa (20,5–48 kPa) in patients with non-alcoholic steatohepatitis, 23,3 kPa (16.1-33kPa) in patients with chronic hepatitis B/C infection and 43 kPa (17.1–75 kPa) in patients with cirrhosis due to autoimmune liver disease.

A total of 14 patients died within the observation period: six patients died prior to FU-1 and eight patients died between FU-1 and FU-2. Death in all 14 patients was not related to COVID-19: three patients died following variceal bleeding, five patients due to acute-on-chronic liver failure (ACLF) and three patients from septic shock. One patient died following perforated sigma diverticulitis, two patients from acute myocardial infarction. Six patients underwent liver transplantation (LT) after enrolment and did not complete FU-2. None of the patients who died or underwent LT before reaching FU-1 was diagnosed positive for SARS-CoV-2 infection or developed COVID-19. A total of 51 patients were lost to follow-up, 109 patients completed FU-1 after 52 days (range: 18–110 days), 72 patients completed FU-2 at a median of 6.1 months (range: 2.1–9.8) after enrolment.

At baseline, 51% (n = 78) individuals were enrolled as inpatients, 49% (n = 70) were recruited at the outpatient department. In LC patients, 42% (n = 45) were classified as CPS A, 42% (n = 45) as CPS B and 17% (n = 18) as CPS C. At baseline, median MELD score was 11 (range: 6–26 points). The MELD score was < 9 in 27% (n = 28) patients, 10–19 in 60% (n = 63) and 20–29 in 13% (n = 14) patients. Most frequent cause of LC was alcoholic liver disease (54%), followed by non-alcoholic steatohepatitis (NASH) or non-alcoholic fatty liver disease (NAFLD) in 15%, LC due to chronic hepatitis C (4%) and autoimmune hepatitis (4%). The most common coexisting diseases were arterial hypertension in 44% (n = 47), followed by diabetes (39%; n = 42) and congestive heart failure (10%; n = 11); 25% (n = 27) were smokers. Charlson Comorbidity Index was 0 in 6% (n = 9) of the patients, 2 in 2% (n = 3), 3 in 27% (n = 41), 4 in 23% (n = 35), 5 in 21% (n = 19) and 38% (n = 43) had an Index ≥ 6 points.

On inclusion, 81% (n = 51) of the inpatients had decompensated LC with presence of ascites (77%), hepatic encephalopathy (23%) and variceal bleeding (2%). All inpatients were tested negative for SARS-CoV-2 infection via qRT-PCR of nasopharyngeal swab. Clinical characteristics are depicted in Table 2.

**Table 2:** Clinical characteristics and laboratory findings at baseline of patients with liver cirrhosis (LC) and/or hepatocellular carcinoma (HCC).

Characteristics	LC only	HCC patients	Total
***n***	108	35	143
**Age (median; min/max)**	61 (19–86)	69 (55–85)	65 (19–97)
**Outpatients**	45 (42%)	25 (71%)	70 (49%)
**Male (n; %)**	67 (63%)	28 (80%)	99 (69%)
**Etiology of LC (n; %)**			
• ALD	56 (54%)	13 (37%)	71 (50%)
• NASH/NAFLD	14 (15%)	4 (11%)	20 (14%)
• HBV	0	0	0
• HCV	4 (4%)	5 (14%)	9 (6%)
• AIH	4 (4%)	1 (3%)	5 (4%)
• PBC	2 (2%)	1 (3%)	3 (2%)
• PSC	4 (4%)	0	4 (3%)
• Other	13 (14%)	5 (14%)	20 (14%)
• Multiple Reason	4 (4%)	0	4 (3%)
• No LC	n.a.	5 (17%)	5 (4%)
**Laboratory data (mean; min/max)**			
• Hb (g/dl)	10.7 (5.6–16.3)	12.5 (8.2–18.4)	11.1 (5.6–18.4)
• WBC (10^9^/l)	6.1 (1.6–14.6)	5.6 (2.8–14.9)	6.0 (1.6–14.6)
• Platelet count (10^9^/l)	142 (22–786)	117 (23–293)	136.8 (21–786)
• Albumin (g/l)	31.3 (10.3–79)	34 (17.6–46)	32.2 (10.3–79)
• Bilirubin (mg/dl)	2.5 (0.3–19.5)	1.6 (0.5–5.5)	2.2 (0.3–19.5)
• AST (U/l)	54.7 (18–456)	54 (25–269)	55 (18–456)
• ALT (U/I)	36.5 (9.0–214)	40.1 (9.0–165)	37.4 (9.0–214)
• CRP (mg/l)	18 (4.0–146)	18.2 (4.0–91)	17.8 (4.0–146)
• INR	1.3 (0.9–2.6)	1.2 (0.9–1.9)	1.3 (0.9–3.0)
**CPS (n; %)**			
• A	43 (41%)	17 (61%)	60 (46%)
• B	42 (42%)	7 (21%)	49 (37%)
• C	18 (17%)	5 (18%)	23 (17%)

Abbreviations: AIH, autoimmune hepatitis; ALD, alcoholic liver disease; ALT, alanine aminotransferase; AST, asparatete aminotransferase; CPS, Child-Pugh score; CRP, c-reactive protein; Hb, haemoglobin; HBV, hepatitis B virus; HCV, hepatitis C virus; MELD, model of end-stage liver disease; NASH, non-alcoholic steatohepatitis; PBC, primary biliary cirrhosis, PSC, primary sclerosing cholangitis, WBC, white blood cell.

Out of the 150 patients enrolled, 35 patients had HCC. Six of these patients had no underlying LC. Median age of HCC patients was 69 years (range: 55–85 years), 80% (n = 28) were male. Tumor stage according to BCLC classification was BCLC A in 32% (n = 9), BCLC B 50% (n = 14) and BCLC C in 18% (n = 5). Treatment modalities included transarterial chemoembolization (TACE) in 75% (n = 18), systemic chemotherapy in 17% (n = 4), including the tyrosine kinase inhibitors sorafenib (n = 3) and lenvatinib plus pembrolizumab (n = 1), microwave ablation in 4% (n = 1) and best supportive care (BSC) in one patient.

**Fig 2:**
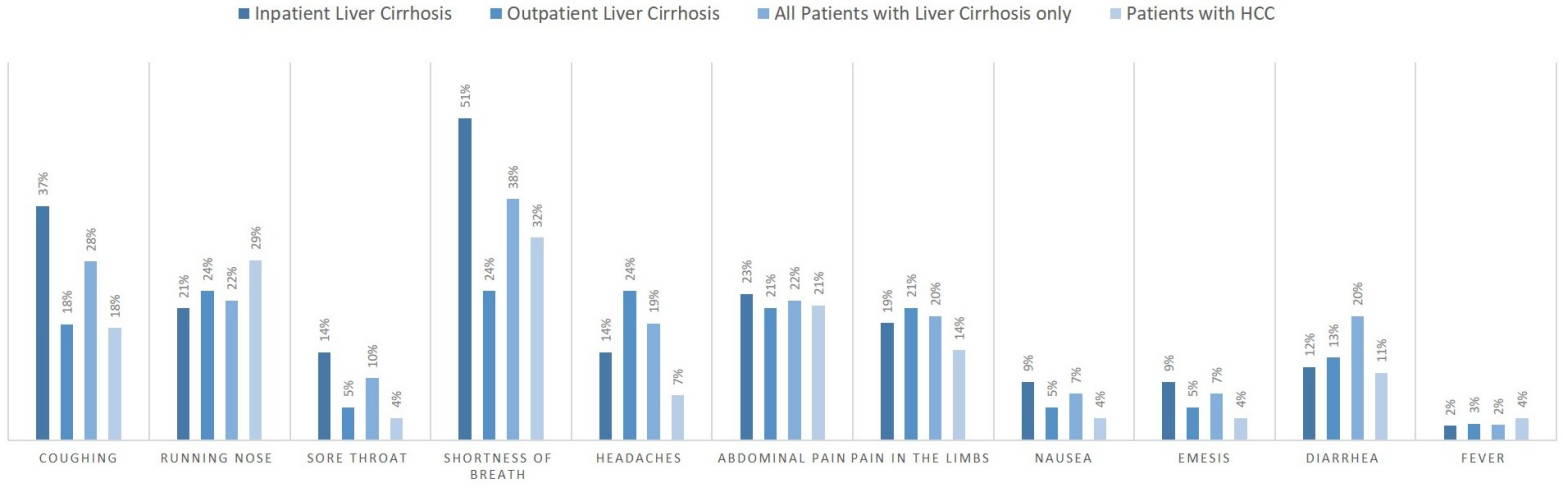
Distribution and frequency of clinical symptoms at baseline. Shortness of breath was the most frequent symptom in all patients, while sore throat (10%) and fever (2%) were only present in a minority of patients.

At baseline, clinical symptoms that were possibly related to COVID-19 were present in 70% of patients (n = 77): shortness of breath n = 40 (37%) and coughing m = 28 (26%) were the most frequent symptoms. Patients who reported shortness of breath had a significantly higher Charlson Comorbidity Index (4 vs. 5 points, *p* = .022), while no significant difference was found in patient with chronic heart insufficiency (6 vs. 0 patients, p = .09), chronic lung disease (4 vs. 6 patients, p = .34) and in patients with coronary heart disease (3 vs. 4 patients, p = .22). Distribution and frequencies of clinical symptoms reported at baseline are depicted in Fig 2.

**Fig 3:**
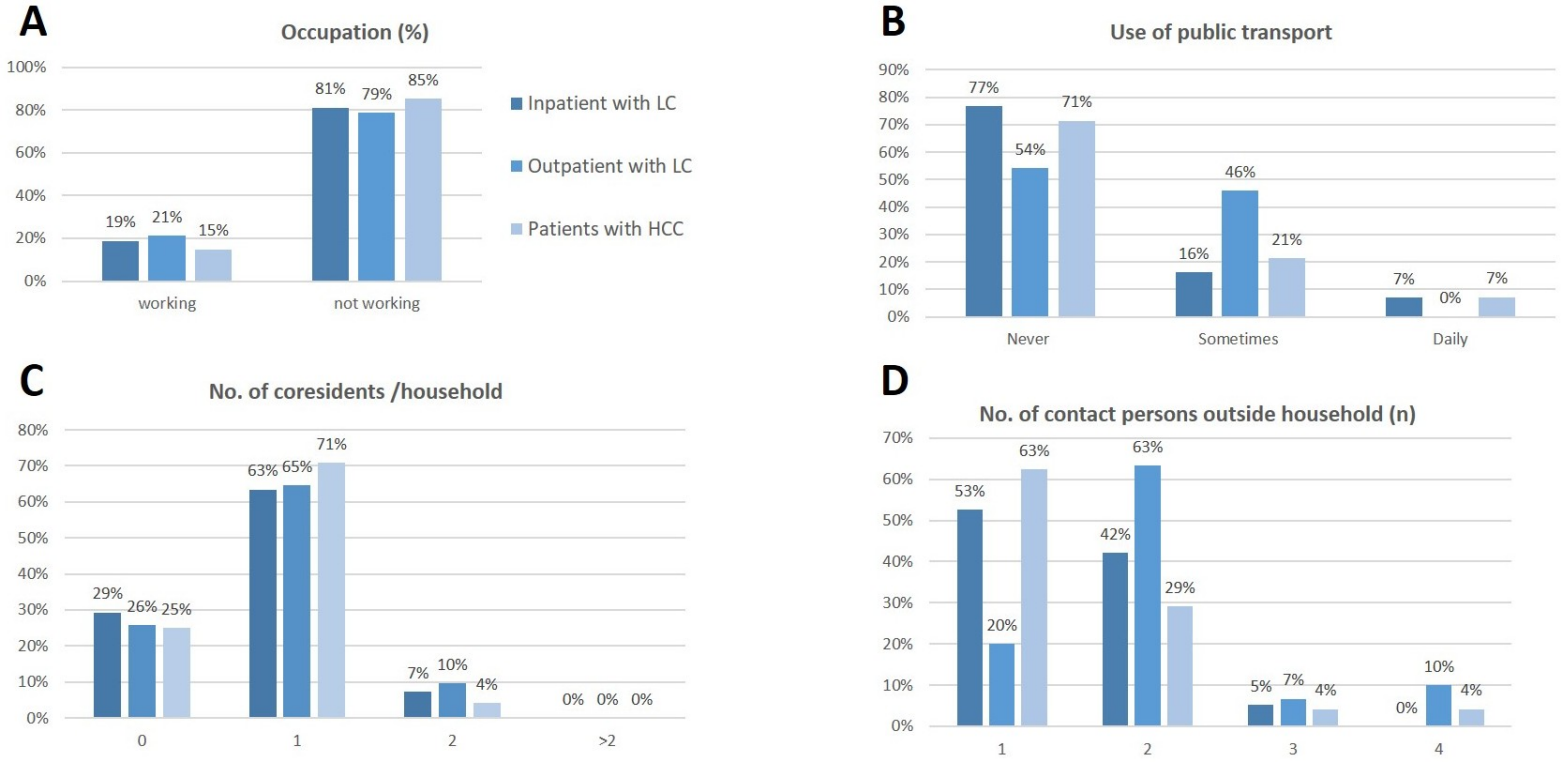
Distribution and frequency of social interactions and risk factors for acquiring viral infection. The majority of patients were not working (A) and only 7% of patients with liver cirrhosis used public transport prior to enrolment (B). Nearly all patients lived together with their spouses or even alone (C), while only 3% of the patients with liver cirrhosis lived together with children in a household. Beside household contacts, patients with HCC had mostly only contact to one person outside the household while patients with liver cirrhosis predominately had contact to two other persons per day. LC: liver cirrhosis.

One patient with LC reported direct contact to a patient with known COVID-19 a baseline, only one out of 109 patients had travelled through Germany within four weeks prior to enrolment, n = 5 (5%) patients reported daily use of public transport while n = 73 (68%) were not using public transport at any time. Thus, risk factors for SARS-CoV-2 exposure were present only in a minority of patients. For distribution of risk factors see Fig 3.

**Fig 4:**
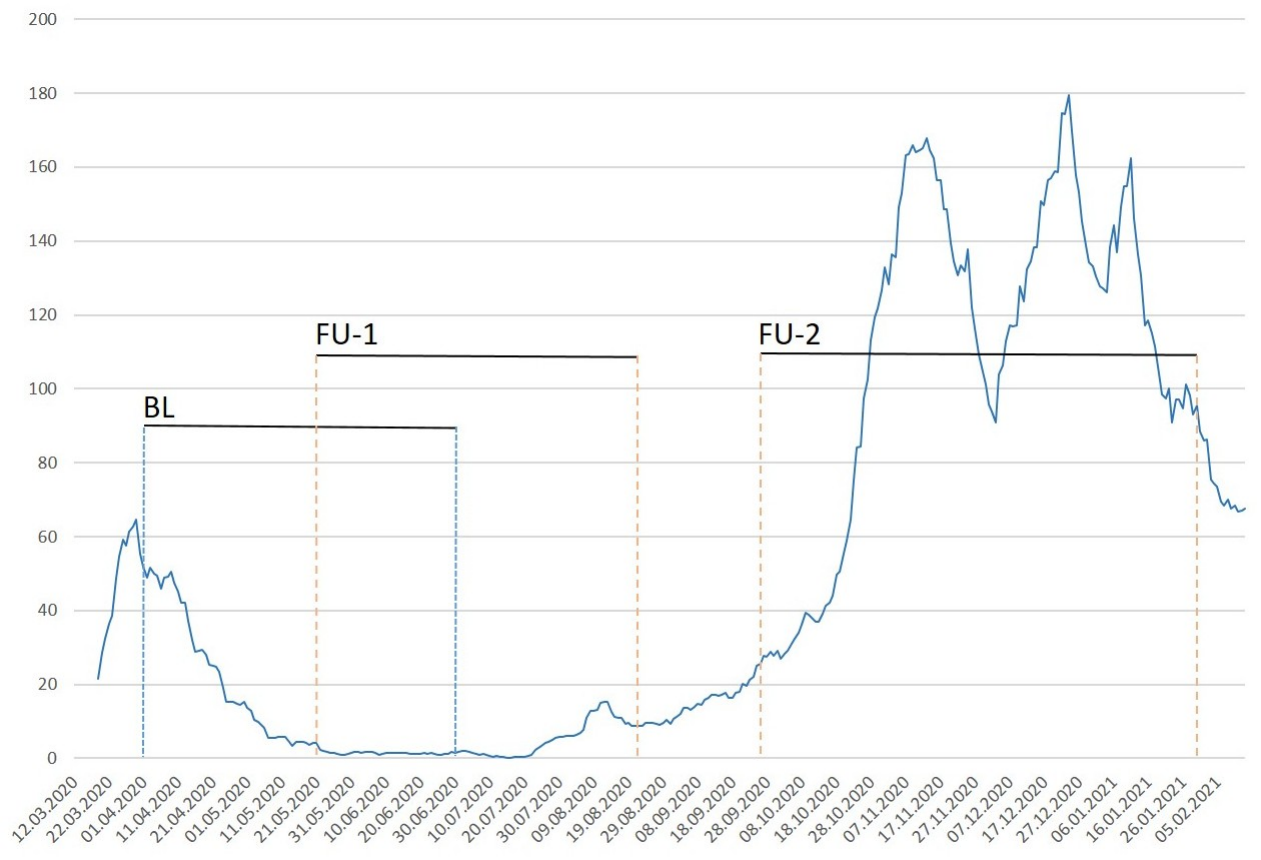
Incidence of SARS-CoV-2 infections at the city of Hamburg during the study. The local incidence of SARS CoV-2 slowly decreases druring the baseline period between 2th April to 30th June and remaind on a low level, when FU-1 was conducted. At the beginning of FU-2, a rapid increase was found with a maximum incidence rate on 24th December 2020. Y-Axis: 7-day incidence rate per 100.000 citizens of Hamburg; BL, baseline; FU-1, first follow-up visit; FU-2, second follow-up visit. Data adapted from: https://www.hamburg.de/corona-zahlen; accessed 14th June 2021.

During the 6 months’ observational period until January 31, 2021, 284 presentations to the outpatient department were assessed in all 109 patients who were eligible for final analysis, accounting for 2.5 visits per patient on average. A total of 33 patients were admitted or readmitted after study inclusion, resulting in 760 hospital days, accounting for an average of 23 hospital days per inpatient. The seven-day incidence rate of SARS-CoV-2 infections at the city of Hamburg varied during the study: While the incidence rate was decreasing from 55 cases per 100.000 citizens at 1th April 2020 to the lowest count 0.9 at 26^th^ of June, rapid increase was found when FU-2 visits were scheduled, beginning in September 2020 (Fig 4).

All patients who had been included during hospitalisation or who had been readmitted were diagnosed PCR-negative for SARS-Cov-2 infection via nasopharyngeal swab on admission or within 5 days prior to admission. At baseline, only one patient out of 143 (0.7%) was tested positive for anti-SARS-CoV2-antibodies, indicating previous infection. This was an 83 years old male with compensated alcoholic LC and HCC, BCLC stage C, treated with lenvatinib and pembrolizumab. Anti SARS-CoV-2 antibodies were detected at baseline and at FU-1 six weeks later. The patient developed tumorprogression soon after FU-1 and died at 28^th^ of September, three months after enrolment.

Of note, this patient has denied any symptoms of COVID-19 in the past and throughout the observational period. In none of the other patients who completed FU-1 (n = 110), antibodies against SARS-CoV-2 virus were detected. At FU-2, conducted after a median of 6.1 months, no further patient (n = 72) was tested positive for SARS-CoV-2 infection. Thus, there was no anti-SARS-CoV2-seroconversion during the observational period.

## Discussion

Preventive restrictions to contain transmission of SARS-CoV2-virus may severely affect standard medical care by interfering with treatment and surveillance routines. In our study, we prospectively evaluated the rate of COVID-19 in a cohort of patients with LC/HCC with physical presence at our medical center, deemed to be at high risk for developing severe COVID-19.

A recent announcement by the EASL and the European Society of Clinical Microbiology and Infectious Diseases (ESCMID) recommends management of LC outpatients to be stratified according to liver status (e.g. decompensated or compensated LC), while care should be maintained in patients with HCC [15, 22]. While data are lacking regarding the incidence of COVID-19 in LC outpatients, the seroprevalance of anti-SARS-CoV-2 antibodies in cancer outpatients was found to be 30% in a recent study, underlining that these patients are potentially more prone for viral infection and have to be protected, when presenting for routine visits [23].

Human-to-human transmission through coughing or sneezing and inhaling infectious respiratory droplets is the primary route of SARS-CoV-2 infection and use of public transport as well as public gathering has been identified early during the outbreak as a source of virus transmission and disease spread [24, 25]. Considering the spatial conditions in hospital waiting rooms, it appears reasonable that the risk of viral infection is elevated due to the relatively close contact to possible SARS-CoV-2 infected patients. Furthermore, direct contact to healthcare workers may also imply an increased risk for viral transmission: Shields et al. report a point prevalence of asymptomatic, SARS-CoV-2 PCR-positive healthcare works (HCW) of 2.4% and Kasztelewitz et al. found, that more than 50% of the HCW, who had been tested positive for SARS-CoV-2 antibodies, denied any clinical symptoms in the past weeks prior testing, illustrating that these HCW had been asymptomatic but possibly infectious at a time [26, 27].

Patients in our study reported a strict avoidance of exposure and adherence to contact restrictions: 75% lived together with only one person in a household, only 5% of all patients used public transport daily, while 68% stated to avoid public transport. Only one patient (0.7%) reported recent travel activities. Furthermore, 45% had contact only to two other persons outside their households. Thus, while social contacts were limited in our cohort, presentation at a university referral center remained a potential risk factor for SARS-CoV-2 infection in our study cohort.

According to the recommendations of the local COVID-19 task force at the University Medical Center, decision was made at the I. Department of Medicine to continue outpatient care for patients with LC and/or HCC under defined preventive measures: in waiting areas, patients were placed with a distance of at least 1.5 meter to each other, patients were questioned for symptoms of viral infection before entering the waiting area and were advised to wear face masks when presenting to the outpatient department. Physicians were advised to wear masks at every patient contact. Preventive measures were first implemented in March 19, 2020, thirteen days before the first patient of this study was enrolled on April 1.

In our study, only one asymptomatic patient was diagnosed positive for SARS-CoV-2 antibodies, while during the observation period with more than 280 contacts to the outpatient clinic, none of the remaining 108 patients developed SARS-CoV-2 infection. It has to be noted that, in addition to the preventive measures at our outpatient clinic, several legal requirements in Germany and the City of Hamburg, such as contact and travel restriction, mandatory wearing of face mask in public transport etc., had been issued during the follow-up period. But in addition to this, the preventive measures that had been implemented at our outpatient department appear to be effective to protect patients from SARS-CoV-2 infection when presenting for routine visits.

The effectiveness of these infection control interventions is further supported by a previous study from our center, documenting SARS-CoV-2 antibodies in only 22 (1.8%) out of 1253 HCW, who had been tested at the University Medical Center Hamburg-Eppendorf between March 20 and July 17 2020 [28]. Similarly, a low seroprevalence of 1,6% among 316 HCW was reported from another German tertiary center at the University Medical Center Essen [29].

Although the overall incidence of COVID-19 in our cohort was low and no subsequent deterioration of liver function was found in the one patient diagnosed positive for SARS-CoV-2 infection, the mortality rate was considerably high: Out of 143 patients analysed, 14 patients (9.7%) died prior to FU-2. While none of these patients had been tested positive for SARS-CoV-2, all patients had advanced liver disease (CPS B and C) and died either because of an acute emergency (upper GI-bleeding, myocardial infarction) or of acute-on-chronic liver failure (ACLF). ACLF is characterized by a high mortality rate of approximately 58% and in patients with LC, the mortality of myocardial infarction is significantly elevated compared to non-cirrhotic patients [29, 30]. Considering this, the mortality in our cohort was related to the underlying LC but not to COVID-19.

It has to be noted that incidence of SARS-CoV-2 in the City of Hamburg varied during the study: reaching a first maximum of 64.5 cases per 100.000 citizens on March 30, the incidence was slightly decreasing to 52/100.000 on April 1, when this study was initiated and the first patient was included. Until June 30, when the last patient was enrolled, the incidence markedly decreased to 2.1/100.000 but then peaked to maximum of 179.6/100.000 at December 24 [31]. The fact that the overall SARS-CoV-2 incidence in the city of Hamburg had decreased constantly during recruitment and FU-1 may have contributed to the comparable low rate of SARS-CoV-2 infections at baseline and FU-1. But despite the rapidly increasing incidence starting at the end of September 2020, which lead to a nationwide second shutdown on December 16. 2020, the incidence rate in our cohort still remained low. This further underlines, that despite a high incidence, medical care appears to be safe and feasible in LC and HCC patients, when preventive measures have been implemented and should therefore be maintained. Still our study has limitations that need to be acknowledged: First, the limited number of patients and the monocentric design may limit the generalisability of our findings, second, compliance with preventive measures was not recorded in our study and third, the effect of anti-SARS-CoV-2-vaccinations, which are broadly available nowadays could not be assessed.

Of note, the majority of individuals in this study reported symptoms resembling COVID-19 but none of these symptomatic patients was tested positive for SARS-CoV-2 infection. While fever, fatigue, dry cough and loss of smell have been reported as the most frequent symptoms in up to 98.6% of COVID-19 patients, patients were additionally questioned for presence of abdominal pain, nausea, emesis and diarrhoea, because these symptoms had been described in COVID-19 patients early during the pandemic [4, 32]. But given the fact that symptoms such as abdominal pain, emesis and nausea might be more frequent in patients with chronic liver disease, especially in patients with decompensated cirrhosis and ascites, their relevance as surrogate for COVID-19 in our study cohort of patients with chronic liver disease is questionable. Shortness of breath, the most frequent symptom, was found significantly more often in patients with a higher Charlson Index, indicating that other comorbidities and underlying frailty may have contributed to the onset of this symptom. Furthermore, fever was only present in 3% of the patients and coughing was also less frequent (26%) in our cohort. Given this discrepancy between the previously described frequency of symptoms in COVID-19 patients and the low prevalence of fever and dry cough in our study with a SARS-CoV-2 infection rate below one percent, a structured symptom-based questionnaire for patients presenting to the outpatient department remains a useful tool for early identification of patients with SARS-CoV-2 infection and prior to prolonged face to face contact with medical staff.

## Conclusion

In our study, the incidence of SARS-CoV-2 infection in LC and HCC patients, who repeatedly presented to a German tertiary medical center and were therefore deemed to be at risk of COVID-19, was considerably low after infection preventive measures had been established early during the pandemic. The overall mortality rate in our cohort was 8.6% and was primarily caused by ACLF or acute emergencies. SARS-CoV-2 infection did not contribute to this mortality rate, since none of the patients who had died in our cohort had been tested positive for SARS-CoV-2 infection.

In summary, if simple preventive measures are implemented at a tertiary center, medical care for patients with LC with and without HCC appears to be safe even in the absence of effective vaccination strategies and should not be withheld during the SARS-CoV-2 pandemic.
